# Nonwoven fabric coated with cerium oxide nanoparticles for viral inactivation and transmission Inhibition

**DOI:** 10.1038/s41598-025-94199-4

**Published:** 2025-03-25

**Authors:** Eisuke Umezawa, Kan Fujino, Hiroko Ito Yamanaka, Shota Sekiguchi, Takahiro Motoshiromizu, Miho Kawanishi Ouchi, Shunsuke Murai, Tomohide Masuda, Masateru Ito, Hitoshi Nobumasa, Satoshi Taharaguchi

**Affiliations:** 1 Laboratory of Microbiology, School of Veterinary Medicine, Azabu University, Sagamihara, Kanagawa Japan; 2 New Frontiers Research Laboratories, Toray Industries, Inc, 6-10-1, Kamakura, 248-8555 Kanagawa Japan

**Keywords:** Cerium oxide nanoparticle, Nanoceria, Antiviral material, Nonwoven fabric, Virology, Antivirals, Nanoparticles

## Abstract

Studies on virus inactivation by metal nanoparticles indicate that antiviral activity is influenced by the stabilizer on the particle surface. Additionally, cerium oxide nanoparticles stabilized with boric acid (BA-CeO_2_) exhibit potent antiviral activity. However, previous studies utilized BA-CeO_2_ dispersed in liquid form and did not fully account for the practical application of antiviral materials in real-world environments. We investigated the antiviral activity of nonwoven fabric coated with BA-CeO_2_ (NC-NWF). When a medium containing viruses was placed on NC-NWF, the titers of mouse hepatitis virus (MHV), influenza A virus, and feline calicivirus were reduced by > 99% within 2 h. Furthermore, the transmission of MHV was assessed in cages lined with NC-NWF. The cages were divided into two compartments using a mesh and NC-NWF, housing infected and uninfected mice on either side. The results indicated a significantly lower antibody titer against MHV in naïve mice with the NC-NWF partition than in the control partition. Additionally, placing infected mice in NC-NWF cages for 2 h, followed by naïve mice for 24 h, resulted in lower antibody titers against MHV than those in the control fabric. These findings suggest that NC-NWF exhibits antiviral activity and retains efficacy in living environments, such as rearing cages.

**Supplementary Information:**

The online version contains supplementary material available at 10.1038/s41598-025-94199-4.

## Introduction

Viral pandemics, such as the Spanish flu and coronavirus disease, periodically occur. Humans will continue to face threats from various viral infections, necessitating ongoing measures to combat them^1,2^. Developing novel vaccines, therapeutics, and other drugs continues to be the primary countermeasure against viral infections. However, considerable challenges remain, including the acquisition of resistance due to viral mutations and the difficulty in developing drugs and vaccines that are effective against a wide range of viral species^3,4^. Extensive research has been conducted on metal-based materials, such as silver and copper; biopolymers, such as chitosan; and materials, such as graphene, all aimed at inactivating viruses^5^. These material-based approaches, particularly those involving metal nanoparticles, face challenges, including difficulties in targeting specific tissues and the accumulation of metal nanoparticles in organs when used for applications, such as post-infection therapy^6^. Nonetheless, these approaches are considered useful because they exhibit antiviral activity against a broad spectrum of viruses and can act on viruses in the environment after their release from the host^5,7,8^.

Silver and copper nanoparticles have been widely studied for their antiviral properties. Silver nanoparticles inhibit viral infection by binding to viral proteins, such as gp120 in HIV-1, preventing interactions with host receptors, such as CD4^9,10^. Additionally, they inhibit replication by interacting with viral particles or DNA in viruses, such as HBV^11^. In contrast, Cu nanoparticles, inactivate viruses by releasing Cu⁺ ions that generate reactive oxygen species, which degrade viral proteins, such as HA and NA in influenza viruses^12^. Recent studies have also highlighted the potential of metal nanoparticles in treatments against emerging pathogens such as SARS-CoV-2. For instance, silver and copper-based nanoparticles have demonstrated potent antiviral effects against SARS-CoV-2, rapidly reducing viral infectivity in vitro^13^. However, there are several issues associated with the use of metal nanoparticles as antiviral materials. In addition to their inherent toxicity to living organisms, concerns include environmental toxicity due to ionic leakage and the high cost of producing high-purity silver and copper nanoparticles. For example, Ag(I) ions released from silver nanoparticles inhibit nuclear receptor-dependent endocrine pathways, such as the thyroid and androgen pathways^14^. Numerous studies have highlighted the reproductive toxicity of metal nanoparticle systems. Silver nanoparticles exhibit concentration-dependent cytotoxicity in C18-4 cells, a spermatogenic stem cell line. They also reduce human sperm motility in a concentration-dependent manner and decrease epididymal sperm counts in rats after intravenous administration^15–17^.

Nanoceria, or cerium oxide nanoparticles, exhibits catalytic activity owing to its ability to alternate between Ce⁴⁺ and Ce³⁺ oxidation states, making it useful in chemical and biological applications. A notable characteristic of nanoceria is its ability to mimic the activities of various enzymes, including superoxide dismutase, catalase, peroxidase, oxidase, and phosphatase^18–20^. Additionally, nanoceria exhibits low toxicity to living organisms compared with that of other metal nanoparticles because of its low solubility, which reduces the release of toxic ions into the environment^15,21,22^. Similar to other metal nanoparticles, nanoceria exhibits antibacterial and antiviral properties and reportedly inactivate various viruses, such as herpes simplex virus, influenza virus, SARS-CoV-2, and transmissible gastroenteritis viruses^23–25^. However, these studies indicate that viral inactivation takes approximately 1 h, with no reports of antiviral activity of < 1 h in these studies.

The antiviral activity of metal nanoparticles is influenced by surface stabilizers. In a previous study, nanoceria was synthesized using citric acid, dextran, polyacrylic acid, polyphosphoric acid, and boric acid as stabilizers, to assess their impacts on antiviral activity^26^. The results indicated that nanoceria with boric acid (BA-CeO_2_) as a stabilizer demonstrated the strongest antiviral activity, reducing the virus titer by over 10³ PFU/mL in 5 min and 10⁴ PFU/mL in 1 h when mixed with influenza A virus (FluA)^26^. However, the rapid and strong antiviral activity of BA-CeO_2_ has been demonstrated only under limited laboratory conditions. To establish the use of BA-CeO_2_ as an antiviral material in living environments, it is necessary to confirm not only the function of BA-CeO_2_ in liquid conditions but also its function as part of other materials, such as nonwoven fabrics used in masks and protective clothing. Recent studies have reported the incorporation of various antiviral nanomaterials into nonwoven fabrics for use in personal protective equipment. The potential applications of antiviral nonwoven fabrics, such as in air filters and public-space usage, are also being explored^27–29^. Therefore, this study investigated the antiviral activity of nonwoven fabric coated with BA-CeO_2_ (NC-NWF) against both experimental and living conditions. Media containing FluA, feline calicivirus (FCV), and mouse hepatitis virus (MHV) were placed on NC-NWF, and the effect on the virus titer was measured. Additionally, NC-NWF was used as bedding in mouse cages to assess its effect on viral transmission from infected to uninfected mice.

## Results

### Nanoceria-coated nonwoven fabrics exhibit antiviral activity

**Fig. 1:**
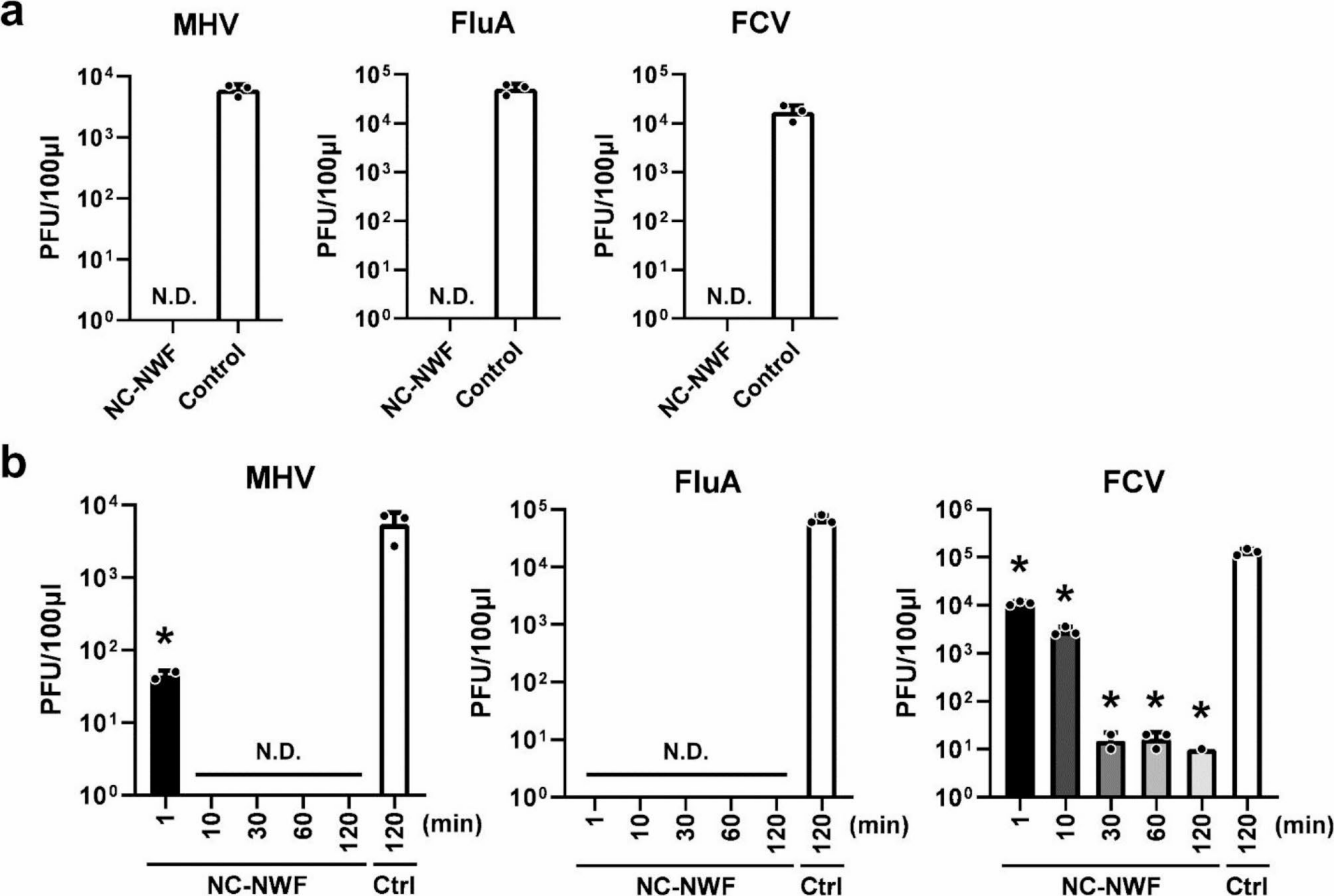
Inactivation of viruses by nonwoven fabrics coated with BA-CeO_2_ (NC-NWF). (**a**) Media containing influenza virus, mouse hepatitis virus (MHV), and feline calicivirus (FCV) were placed on NC-NWF or uncoated control nonwoven fabrics and collected after 2 h to measure virus titers according to ISO 18,184 guidelines. (**b**) Media containing influenza virus, MHV, and FCV were placed on NC-NWF or uncoated control nonwoven fabrics, collected at each time point, and virus titers were measured. The bars represent the mean viral titer observed after each incubation time from three independent experiments. Error bars indicate the standard deviation. Viral titers were measured using the plaque assay method. Statistical significance was analyzed using the Mann–Whitney U test or one-way ANOVA followed by Dunnett’s multiple comparison test, with *p* < 0.05 considered significant. *p < 0.05 (MHV Control [Ctrl] vs. 1 min: *p* = 1.41 × 10^− 8^; FCV Ctrl vs. 1 min: *p* = 2.62 × 10^−7^; FCV Ctrl vs. 10 min: *p* = 1.85 × 10^−9^; FCV Ctrl vs. 30 min: *p* = 5.10 × 10^−14^; FCV Ctrl vs. 60 min: *p* = 7.00 × 10^−14^; FCV Ctrl vs. 120 min: *p* = 3.80 × 10^−14^). ND indicates “not detected” (below the detection limit of 10 PFU/100 µL).

First, the nonwoven fabric was coated with nanoparticles. The BA-CeO₂ nanoparticles used in this study had a Stokes diameter of 5.6 ± 1.6 nm and a zeta potential of + 53 ± 2.0 mV at pH 4.5. A nylon binder, a melamine cross-linker, and cetylpyridinium chloride (CPC) were used to facilitate uniform adhesion of the nanoparticles to the polypropylene nonwoven fabric. As a result, we successfully prepared the nanoparticle-coated nonwoven fabric, referred to as NC-NWF. Next, to investigate the antiviral activity of NC-NWF, experiments were conducted according to ISO 18,184, the standard method for the antiviral testing of textile products. Media containing MHV, FluA, and FCV were applied to NC-NWF, and viral titers were measured using a plaque assay after 2 h of incubation at 25 °C (Fig. 1a). The viral titers were below the detection limit for all three viruses, with at least a 10^2.87^ PFU/mL reduction for MHV, 10^3.7^ PFU/mL for FluA, and 10^3.12^ PFU/mL for FCV, compared to that of the control nonwoven fabric. NC-NWF effectively reduced the viral titers of all tested viruses, with a > 99% reduction after 2 h (Fig. 1a). These results confirm the strong inactivating effect of NC-NWF on enveloped and non-enveloped viruses. Subsequently, viral titers were measured over time to determine the time required for NC-NWF to inactivate the viruses (Fig. 1b). Suppression of > 10⁴ PFU/mL was observed at 1 min and the titers were below the detection limit at 10 min for FluA and MHV. A reduction of approximately 10⁴ PFU/mL was observed within 30 min for FCV; however, even after 2 h, 10 PFU/mL of the virus was still detected in a single trial. These results suggest that NC-NWF can rapidly and effectively inactivate viruses, particularly enveloped viruses. Additionally, to assess the influence of the coating agent alone (i.e., without BA-CeO_2_), we performed antiviral activity tests using FCV and FluA. We compared nonwoven fabrics treated only with the coating agent to untreated (uncoated) nonwoven fabrics, finding no significant differences in FCV viral titers (*p* = 0.274; Fig. S1a). Conversely, although a statistically significant reduction in FluA titer was observed (1 min: *p* = 5.00 × 10⁻⁶; Fig. S1b), the decrease was limited to approximately 10² PFU/mL at 1 min, and the titer remained above the detection limit even after 120 min. This contrasts with the marked reduction observed when BA-CeO_2_ was added (Fig. 1b). These findings indicate that the coating agent used in this study did not exhibit any antiviral activity against FCV when used alone. Furthermore, although the coating agent itself exhibited a baseline antiviral effect against FluA, removing BA-CeO_2_ markedly reduced this effect, thereby confirming that BA-CeO_2_ contributes substantially to the overall antiviral efficacy against FluA.

### BA-CeO_2_ exhibits low levels of acute toxicity (GHS category 4) in mice

NC-NWF demonstrated antiviral activity in an experimental environment. Then, we evaluated the potential toxicity of BA-CeO_2_ using standard Organization for Economic Co-operation and Development (OECD) guidelines. Specifically, we performed TG423 (Acute Toxicity, Oral), TG473 (Chromosome Aberration), TG406 (Skin Sensitization), TG404 (Skin Irritation), and TG405 (Eye Irritation) tests. The TG423 results indicated relatively low toxicity, classified as GHS Category 4 (oral LD50 value ranging from 300 mg/kg to 2,000 mg/kg) or above. All other tests yielded negative results (Table 1).

**Table 1:** Results of toxicity tests on the nanoparticles.

Test Item	Test method	Result
Acute toxicity (Oral)	OECD TG423	GHS Category 4 or above
Chromosome aberration	OECD TG473	Negative
Skin sensitization	OECD TG406	Negative
Skin irritation	OECD TG404	Negative
Eye irritation	OECD TG405	Negative

### NC-NWF partition inhibits MHV transmission in a compartmentalized living environment

**Fig. 2:**
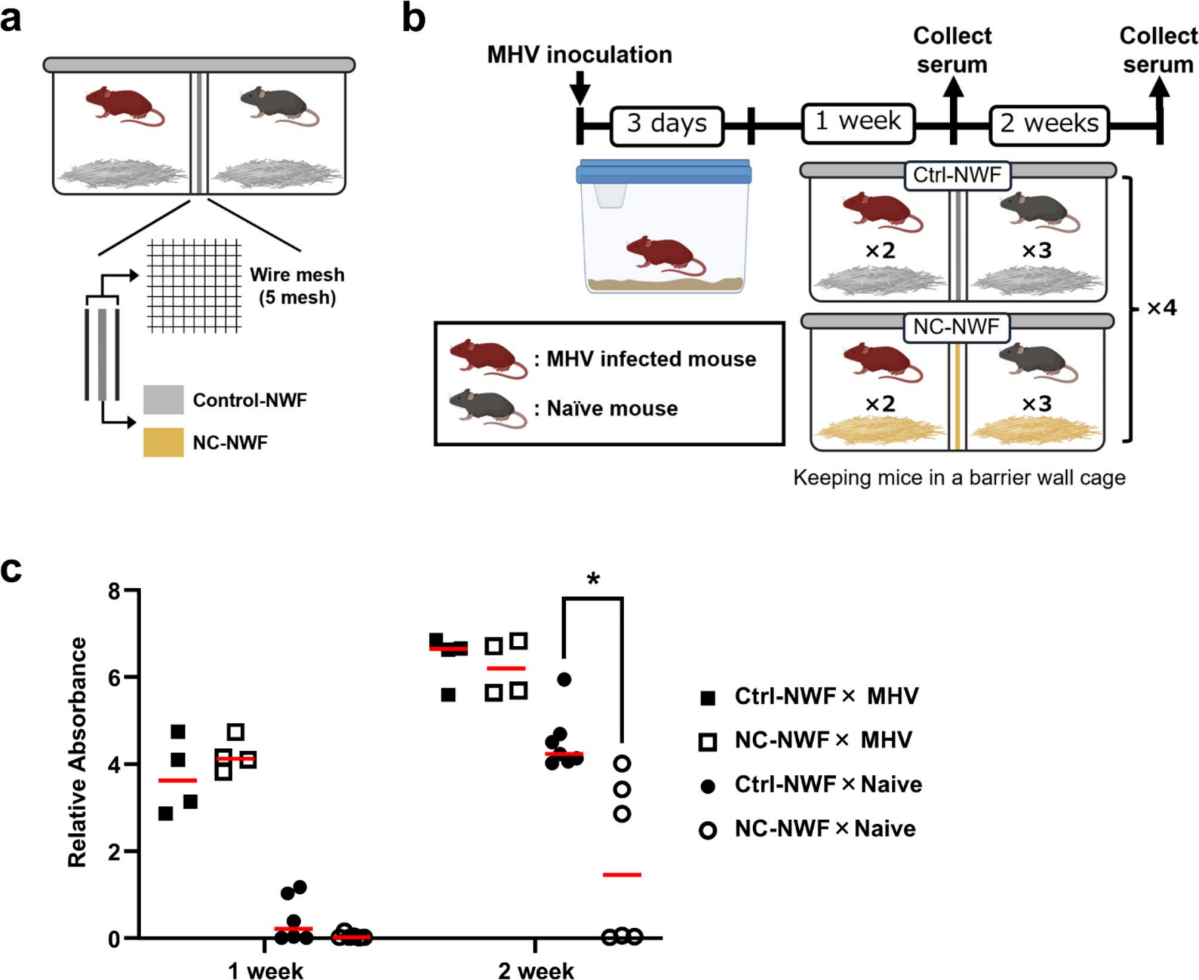
NC-NWF inhibits the transmission of infection during cohabitation with mice infected with MHV. (**a**,**b**) Schematic showing the cage used in the experiment and the experimental schedule. The cage was bisected by metal mesh as shown in the figure, and the walls and floor were covered with either NC-NWF or uncoated control nonwoven fabric. Created in BioRender. Fujino, K. (2024) BioRender.com/r31r614 and BioRender.com/l50d839. (**c**) MHV-infected and -naïve mice were co-housed, and sera were collected at 1 and 2 weeks to measure MHV antibody titers. The vertical axis represents the measured absorbance (A492nm - A610nm), which has been corrected using the positive control supplied with the kit. Statistical analyses were conducted using a multiple Mann–Whitney test, with *p* < 0.05 considered significant (2 weeks: *p* = 1.17 × 10^−3^). MHV, mouse hepatitis virus; NC-NWF, nonwoven fabrics coated with BA-CeO_2_.

Based on these toxicity assessments, we conducted a partition experiment using mice to confirm whether NC-NWF exhibits antiviral activity in a living environment. The bedding in the mouse cages consisted of either NC-NWF or a control nonwoven fabric. The cages were further divided into two compartments using a metal mesh and their respective nonwoven fabrics (Fig. 2a). Additionally, the floors of the cages were covered with shredded pieces of the respective nonwoven fabric as bedding. Mice inoculated with MHV were housed in one compartment, while uninfected (naïve) mice were housed in the other compartment. Sera were collected on days 7 and 14 to measure the MHV antibody titers (Fig. 2b). On day 14, antibody titers in naïve mice were significantly lower (*p* = 1.17 × 10^−3^) with NC-NWF as the lining and bedding than with the control nonwoven fabric (Fig. 2c).

### NC-NWF inhibits MHV transmission through contaminated bedding

**Fig. 3:**
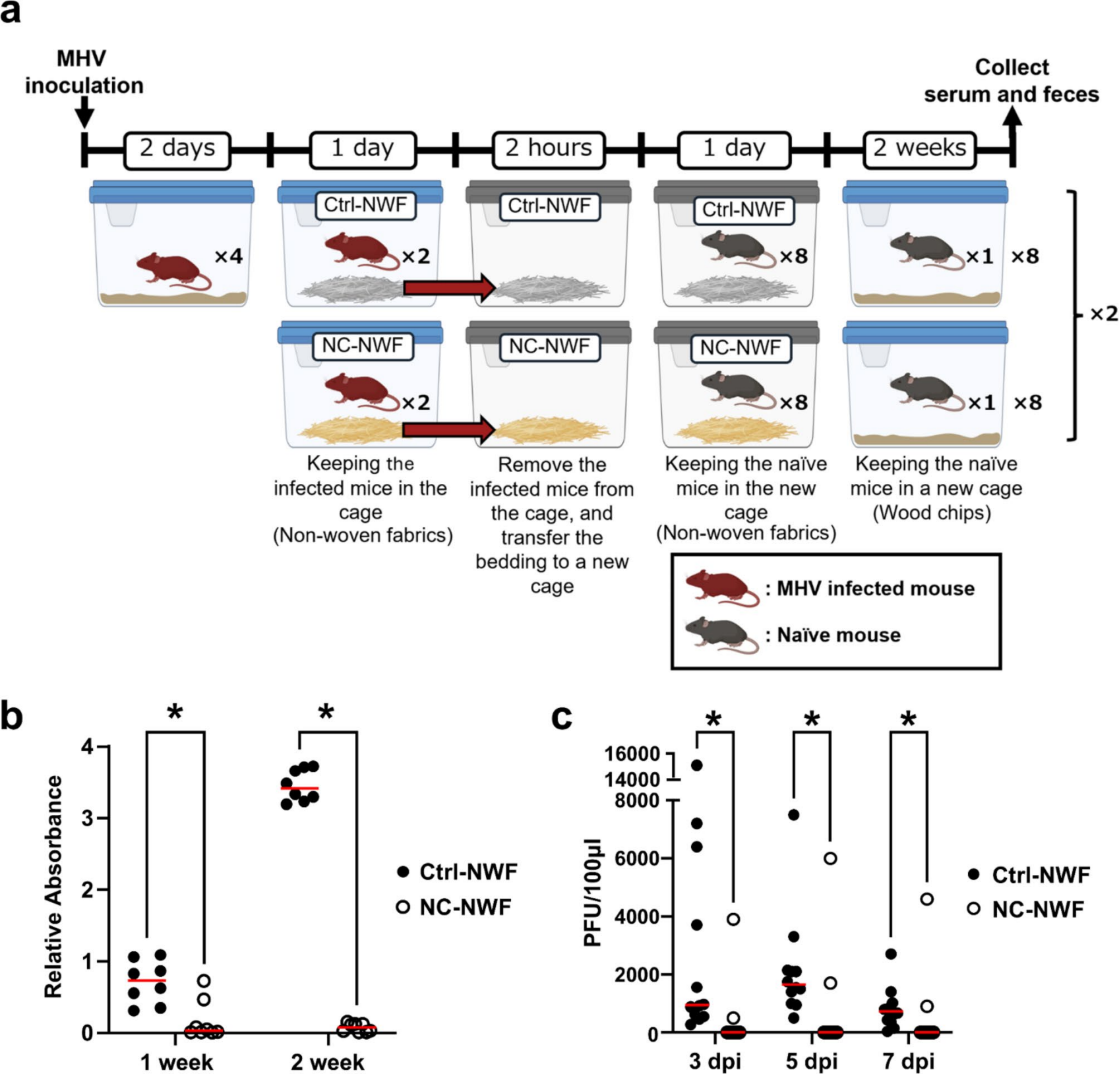
NC-NWF inhibits the transmission of infection by an environment contaminated by mice infected with MHV. (**a**) Schematic showing the cage used in the experiment and the experimental schedule. Created in BioRender. Fujino, K. (2024) BioRender.com/b23u025. (**b**,**c**) Infected mice were placed in the cage and removed 24 h later, and naïve mice were introduced 2 h later. After 24 h, the mice were removed and divided into NC-NWF and control groups. MHV antibody titers (**b**) and MHV viral titers (**c**) were measured 7 and 14 d later. The vertical axis represents the measured absorbance (A492nm - A610nm), which has been corrected using the positive control supplied with the kit. Statistical analyses were performed using a multiple Mann–Whitney test, with *p* < 0.05 considered significant (1 week: *p* = 1.09 × 10^−3^; 2 weeks: *p* = 1.55 × 10^−4^; 3 dpi: *p* = 8.30 × 10^−5^; 5 dpi: *p* = 5.28 × 10^−4^; 7 dpi: *p* = 1.51 × 10^−3^). MHV, mouse hepatitis virus; NC-NWF, nonwoven fabrics coated with BA-CeO_2_.

A partition experiment in the same cage as the infected mice demonstrated that NC-NWF could inhibit the spread of MHV. Subsequently, we conducted a switching experiment to confirm antiviral activity in environments contaminated by infected mice. Two days after inoculation with MHV, an infected mouse was placed in a cage with bedding composed of either NC-NWF or a control uncoated nonwoven fabric. After 1 d, the infected mouse was removed, and the bedding was transferred to a new cage. Two hours later, a naïve mouse was introduced into a new cage and maintained there for 24 h. Naïve mice were then housed individually. Antibody titers were measured on days 7 and 14, whereas viral titers in the feces were measured on days 3, 5, and 7 (Fig. 3a). The viral antibody titer of naïve mice in the NC-NWF group was significantly lower (*p* = 1.09 × 10^−3^ at 1 week; *p* = 1.55 × 10^−4^ at 2 weeks) than that of mice with the uncoated nonwoven fabric (Fig. 3b). Similarly, the viral titers in the feces were significantly lower (3 dpi: *p* = 8.30 × 10^−5^; 5 dpi: *p* = 5.28 × 10^−4^; 7 dpi: *p* = 1.51 × 10^−3^) when NC-NWF was used (Fig. 3c).

## Discussion

In this study, we revealed the antiviral activity of NC-NWF and demonstrated that NC-NWF inhibited the transmission of MHV in the living environment of mice. Our previous study confirmed that nanoparticles dispersed in liquid can inactivate the virus within 5 min of exposure^26^. The findings of this study indicate that NC-NWF exhibits strong antiviral activity comparable to that of nanoparticles dispersed in liquid. Specifically, NC-NWF strongly inhibited (> 99%) the enveloped viruses, FluA and MHV, after 1 min of treatment. However, it required 30 min to inhibit the non-enveloped virus FCV by 99% (Fig. 1). Previous studies investigating the antiviral activity of nanoceria against both enveloped and non-enveloped viruses have also reported that non-enveloped viruses exhibit low susceptibility^23^. The exact mechanism responsible for this difference is unclear and remains to be elucidated. CeO_2_ inactivates viruses by directly binding to the viral envelope and disrupting the lipid bilayer^30^. Additionally, the high binding affinity of nanoceria for viral nucleic acids may play a role in inactivation^23,31^. The exact mechanism of viral inactivation by NC-NWF remains unclear. Our previous study suggests that nanoceria’s antiviral activity is primarily due to the strong adsorption of positively charged BA-CeO_2_ particles onto viruses via electrostatic interactions, as well as the oxidation of viral envelopes and proteins by CeO₂ itself^26^. However, we cannot entirely rule out a contribution from the coating agent, which contains CPC. This compound can disrupt lipid bilayers and thus may assist in inactivating enveloped viruses. To gain a more comprehensive understanding of these processes, it will be crucial to use electron microscopy to visualize direct interactions or structural disruptions of the virus using electron microscopy, and identify any viral proteins undergoing degradation or conformational changes using western blotting. Together, these microscopic and biochemical approaches will help clarify the respective roles of BA-CeO_2_ in antiviral inactivation and advance our knowledge of the underlying mechanism.

Experiments using MHV-infected and uninfected mice demonstrated that NC-NWF inhibited transmission through cohabitation (Fig. 2) and via fomites within the bedding (Fig. 3). MHV is transmitted through airborne aerosols and droplets, direct contact, and indirect contact via fomites^32^. MHV is highly contagious and is known to spread from one cage to another. In the control nonwoven fabric group, antibodies were detected 7 d after cohabitation, and by day 14, antibodies were present in all naïve mice (Fig. 2). Previous reports have indicated that naïve mice introduced into an infected population seroconverted within 14 d^33^. This study confirmed an increase in antibody titers, which is consistent with these findings. Conversely, in the NC-NWF group, after two weeks of cohabitation, three out of the six naïve mice remained antibody-negative, suggesting that the virus was rapidly inactivated, thereby suppressing or delaying infection. Additionally, in the switching experiments, NC-NWF inhibited the transmission of MHV compared to the control nonwoven fabrics (Fig. 3). During the SARS-CoV-2 pandemic, infectious viruses shed by infected individuals are considered to remain in environments that are accessible to touch, causing contact transmission and leading to numerous infections^34^. The results of this study suggest that NC-NWF can inactivate viruses on surfaces after the attachment of droplets or droplet nuclei containing viruses excreted by infected individuals.

To confirm antiviral performance in a mouse housing environment in this study, the cage was separated by walls made of nonwoven fabric and a metal mesh, and the transmission of MHV was examined. The results showed that separating the cage with NC-NWF resulted in significantly lower antibody titers in naïve mice than in the control nonwoven fabric. This suggests that the antiviral effects were sustained in the mouse housing environment for more than a week. Metal nanoparticles are known to form a protein corona by adsorbing proteins, which can reportedly affect the interactions between metal nanoparticles and other substances^35^. The antibacterial activity of silver nanoparticles is reportedly attenuated by protein loading, such as serum and bovine serum albumin, depending on the capping material^36^. However, in our previous study, BA-CeO_2_ in liquid also demonstrated antiviral activity under bovine serum albumin-loading conditions^26^. These findings indicate that NC-NWF may retain its functionality in a living environment containing certain proteins and other contaminants. However, under persistent cohabitation conditions, infection cannot be completely prevented using NC-NWF, because the virus is continuously released, and the animals continue to be exposed to the virus throughout the rearing period. Even if the virus is inactivated by nanoceria, completely suppressing the infection is challenging.

One limitation of this study is that we did not fully isolate the effect of the coating agent itself (which contains CPC) from that of BA-CeO_2_. During NC-NWF fabrication, CPC plays the essential role of a surfactant to facilitate the impregnation of BA‐CeO_2_ into the nonwoven fabric, which is an unavoidable step. While this reduction was less pronounced than that observed when BA-CeO_2_ was incorporated, the coating agent alone could still significantly reduce FluA titers (Fig. S1b). Consequently, some of the antiviral activity of NC‐NWF, particularly against enveloped viruses, is inherently attributable to the coating agent. However, experiments with FCV demonstrated that the coating agent alone did not produce a significant antiviral effect (Fig. S1a), and its efficacy against FluA remained inferior to that of NC‐NWF. BA-CeO_2_ itself reportedly inactivates enveloped viruses effectively^26^ and showed antiviral activity against the non-enveloped FCV in this study. Therefore, our findings support the hypothesis that BA-CeO_2_ is likely the main driver of the observed antiviral effects. Future studies should include additional in vitro assays with enveloped viruses, including MHV, and where feasible, in vivo controls to definitively isolate the roles of BA-CeO_2_ and the coating agent. This will help elucidate the potential synergistic or independent effects of the coating components on a broader range of viruses. Although this study evaluated the short-term antiviral efficacy of NC-NWF in a mouse cage environment, we did not assess its durability or antiviral performance under prolonged exposure to heat, humidity, and UV light, nor did we investigate long-term toxicity or potential nanoparticle detachment over extended use. Future research should address these aspects to confirm the long-term safety and sustained antiviral functionality of NC-NWF in real-world settings.

In conclusion, BA-CeO_2_ exhibited strong and rapid antiviral activity even when coated on nonwoven fabrics, effectively inactivating FluA and MHV, and significantly reducing FCV titer. Additionally, NC-NWF demonstrated strong antiviral activity in the living environment of mice and effectively inhibited MHV transmission. CeO_2_ nanoparticles have low solubility and release minimal amounts of cerium ions, contributing to their relatively low biotoxicity and environmental toxicity^22^. Indeed, in this study, standardized OECD guideline tests using mice confirmed that BA-CeO_2_ shows minimal risk (Table 1), consistent with previous reports of the favorable safety profile of CeO₂. These findings support the feasibility of using NC-NWF in environments frequented by humans or animals, as the coating retains its antiviral performance without significant safety concerns. Therefore, NC-NWF may be a valuable antiviral agent in settings that involve frequent contact with living organisms.

## Methods

### Preparation of cells and viruses

Crandell–Rees Feline Kidney (JCRB9035)^37^, Madin–Darby Canine Kidney (IFO50071)^38^, and Delayed Brain Tumor (JCRB1580)^39^ cells were obtained from the Japanese Collection of Research Bioresources Cell Bank, part of the National Institutes of Biomedical Innovation, Health and Nutrition in Japan. The cells were cultured in Dulbecco’s modified Eagle medium (DMEM) (FUJIFILM Wako, Osaka, Japan) supplemented with 10% fetal bovine serum, L-glutamine, and an antibiotic mixture (penicillin 20 IU/mL, streptomycin 0.1 mg/mL) (FUJIFILM Wako), and maintained at 37 °C in a humidified atmosphere with 5% CO₂. The cells were subcultured every 2–3 days to maintain optimal growth conditions. The maintenance medium was DMEM supplemented with L-glutamine and an antibiotic mixture (penicillin 20 IU/mL, streptomycin 0.1 mg/mL). The FluA (strain A/Puerto Rico/8/1934 H1N1: PR8) was kindly provided by Prof. Sakoda at Hokkaido University, while the MHV Y strain was generously provided by Prof. Susan R. at Yale University. Additionally, the FCV F9 strain (VR782) was purchased from the American Type Culture Collection. The viruses used in each experiment were propagated in their respective susceptible cells, and their infectivity and viral titers were assessed using plaque assays.

### Plaque assays

Plaque assays were performed using standard methods^40^. Briefly, cells were seeded in 6-well plates, and upon reaching confluency, they were inoculated with a 10-fold serial dilution of the virus in maintenance medium. After incubation at 37 °C for 1 h, the unbound virus particles were removed by washing the samples with maintenance medium, and an overlay of maintenance medium containing agar was applied. Three days post-inoculation, the agar overlay was removed and viable cells were stained with crystal violet to visualize and count plaques, which were used to calculate the viral titer.

### Investigation of antiviral activity on nonwoven fabrics

Nanoceria with boric acid as a stabilizer (BA-CeO_2_) was synthesized as follows: First, 2.84 g of boric acid was dissolved in 500 mL of water, and sodium hydroxide was added until the solution reached pH 8. Ten milliliters of a 10% Ce(NO_3_)_3_·6H_2_O solution was added, and the solution was stirred for 10 min at 25 °C. Subsequently, 10 g of 1.2% hydrogen peroxide was added dropwise, and the mixture was stirred overnight at 25 °C. Nitric acid solution (5 M) was added until the solution reached a pH of 3. After purification by ultrafiltration (10 kDa), the solution was hydrothermally treated (120 °C, 20 min) to obtain a BA-CeO_2_ dispersion^26^. After synthesis, the dispersion underwent another round of ultrafiltration (5 kDa) to eliminate unreacted components and concentrate the nanoceria. A transmission electron microscopy image of the nanoparticles is shown below, showing their morphology as secondary particles consisting of 2–3-nm primary particles (Fig. S2)^26^. The coating solution contained 1.6 wt% BA-CeO_2_, 0.4 wt% nylon binder, 0.5 wt% melamine cross-linker, and 0.05 wt% CPC at final concentrations. A nylon binder was used to anchor the nanoparticles to the nonwoven fabric and to function as a three-dimensional cross-linker, while a melamine cross-linker was employed to solidify the coating layer. Additionally, CPC was applied to enhance the infiltration of the nanoparticles and coating agent into the nonwoven fabric. Polypropylene nonwoven fabrics were dip-coated with this solution and dried at 130 °C, resulting in BA-CeO_2_-coated nonwoven fabrics. The coating process increased the weight of the nonwoven fabric by 6.92 g/m^2^, incorporating BA-CeO_2_, nylon binder, melamine cross-linker, and CPC. Antiviral activity was assessed according to ISO 18,184 guidelines. The coated nonwoven fabric was cut into 2 × 2 cm pieces with a total weight of 0.4 g and 200 µL of viral suspension was applied. Following an incubation period ranging from 1 to 120 min, the virus was eluted using a 20 mL Soybean-Casein Digest Broth with Lecithin & Polysorbate (FUJIFILM Wako). A 10-fold serial dilution was made in a minimal medium, and the virus was inoculated into the cells, followed by a 2-d incubation period. The viral titer was measured using a plaque assay. The nonwoven fabric before coating was used as the control.

### Animals, virus preparation, and anesthesia

Four-week-old MHV-free female C57BL/6J (B6) mice were purchased from SLC Japan (Hamamatsu, Japan) and acclimatized for one week before the experiments. Mice were housed in autoclaved cages with autoclaved bedding and water bottles. They were provided standard laboratory chow (CLEA Japan, Fujinomiya, Japan) and either distilled or chlorinated tap water *ad libitum*. The rearing conditions were maintained at 22 ± 2 °C under a 12-hour light/dark cycle. All procedures, including anesthesia, viral inoculation, and sampling, were performed by veterinarians in compliance with the 3Rs (replacement, reduction, and refinement). The animals’ conditions were meticulously monitored by veterinarians throughout the experimental period. The MHV was prepared from liver homogenates at a concentration of 3.4 × 10³ PFU/100 µL. Viral inoculation was performed under anesthesia. For anesthesia, 0.75 mL of dexmedetomidine hydrochloride (1 mg/mL) (NIPPON ZENYAKU KOGYO CO., LTD., Fukushima, Japan), 2 mL of midazolam (5 mg/mL) (Novartis, Basel, Switzerland), and 2.5 mL of butorphanol tartrate (5 mg/mL) (Meiji Seika Pharma Co., Ltd., Tokyo, Japan) were mixed in 19.75 mL of water, and 0.01 mL/10 g was intraperitoneally administered^41^. To recover from anesthesia, atipamezole (5 mg/mL) (Kyoritsu Seiyaku Corporation, Tokyo, Japan) was intraperitoneally administered at 0.01 mL/10 g after viral inoculation. Sodium pentobarbital was used to induce deep anesthesia for heart blood collection through cardiac puncture and necropsy procedures. Euthanasia was performed by exsanguination under deep anesthesia to minimize pain and distress, following the ARRIVE guidelines. The solution was prepared by dissolving 3 g of sodium pentobarbital (NACALAI TESQUE, INC., Kyoto, Japan) in 5 mL 95% ethanol, followed by the addition of 15 mL 0.9% saline and 20 mL propylene glycol. The solution was brought to a final volume of 50 mL with 0.9% saline and filtered through a 0.2-µm filter. Pentobarbital (60 mg/mL) was intraperitoneally inoculated at 0.01 mL/10 g. All animal experiments were reviewed and approved by the Animal Experiment Committee of Azabu University (Approval No. 220322-3) and conducted in accordance with the Azabu University Animal Experiment Implementation Regulations and the Animal Experiment Implementation Manual. The animal experiments conducted in this study were reported in accordance with the ARRIVE guidelines (https://arriveguidelines.org), ensuring transparent and accurate reporting of animal research.

### Partition experiment

Eight mice were inoculated with MHV as described above and maintained in cages for 3 d. A plastic two-compartment mouse-rearing cage (TM-TPX-10-WS; Tokiwa Scientific Instruments, Tokyo, Japan) with a partition was used in this experiment. Each compartment of the cage was filled with approximately 50 g of shredded nonwoven fabric (Ctrl-NWF or NC-NWF) as bedding. Additionally, the partitions were made of nonwoven fabric (Ctrl-NWF or NC-NWF) sandwiched between stainless-steel meshes to avoid direct contact between the mice. In each experimental unit, three naïve (MHV-free) mice were introduced into one compartment and two MHV-infected mice were introduced into the other compartment, forming a total of five mice per unit. Each group (Ctrl-NWF and NC-NWF) consisted of four experimental units, resulting in 12 naïve and eight infected mice per group. To assess progression over time, six naïve mice from each group were euthanized and sampled after one week of cohabitation, and the remaining six naïve mice were euthanized and sampled after two weeks. Blood samples were collected using cardiac puncture at the time of euthanasia. For the mice that remained until the two-week time point, the bedding was replaced with fresh nonwoven fabric of the same type on day 7 to maintain appropriate rearing conditions.

### Switching experiment

Eight mice were inoculated with MHV as described above and kept in cages for 2 d. Plastic mouse-rearing cages (218 W × 320 D × 133 H mm^3^) were used for the switching experiment. Approximately 100 g of finely cut nonwoven fabric (Ctrl-NWF or NC-NWF) was used as bedding material in each cage. Two mice inoculated with the MHV were introduced into each cage. Briefly, 24 h after introduction, the MHV-infected mice were removed from their cages, and the bedding was transferred to a new cage. After 2 h, eight naïve mice were introduced into a new cage and maintained there for 24 h. After 24 h, each naïve mouse was individually housed in a separate cage. The above system formed a set, with two sets allocated to the ctrl-NWF group and another two sets allocated to the NC-NWF group. Blood samples were collected via cardiac blood sampling for 7–14 d. Fecal samples were collected from 12 mice in each group after 3, 5, and 7 d and stored at − 80 °C.

### Measurement of anti-MHV antibody titer

For blood sampling, deep anesthesia was induced using intraperitoneal pentobarbital at 0.01 mL/10 g. Once deep anesthesia was confirmed, a cardiac puncture was performed to collect 0.3–0.6 mL of blood, followed by cervical dislocation. Serum was separated by incubating the blood at 37 °C for 30 min, followed by overnight storage at 4 °C. Samples were centrifuged at 1500 ×*g* for 10 min, and serum was collected and stored at -20 °C until ELISA analysis^42^. The anti-MHV antibody titer was measured using a MONILIZA MHV antibody ELISA kit (WAKAMOTO PHARMACEUTICAL CO., LTD., Tokyo, Japan), following the manufacturer’s protocol.

### Measurement of MHV viral titer

Fecal samples were collected and weighed at each time point. DMEM containing antibiotics (penicillin 200 IU/mL, streptomycin 1 mg/mL) was added to create a 10% emulsion, and fecal samples were homogenized at 3000 rpm for 30 s using a homogenizer and stainless-steel beads. After homogenization, the supernatant was collected using centrifugation at 3000 ×*g* for 5 min at 4 °C. The collected supernatant was serially diluted 10-fold and inoculated into DBT cells, and the viral titer was determined using a plaque assay.

### Statistical analysis

All statistical analyses were performed using GraphPad Prism. For the in vitro experiments, viral titers were compared between the NC-NWF and control groups using the Mann–Whitney U test, while differences among multiple groups were evaluated using one-way ANOVA followed by Dunnett’s multiple comparison test to compare the control group with each treatment group. In both analyses, values below the detection limit (< 10 PFU/100 µL) were assigned a placeholder value of 10 PFU/100 µL for statistical analysis. For the in vivo experiments, statistical significance was determined using one-way ANOVA or multiple Mann–Whitney test, and *P* < 0.05 was considered as statistically significant. An asterisk (*) indicates a statistically significant difference (* *P* < 0.05).

## Electronic supplementary material

Below is the link to the electronic supplementary material.

Supplementary Material 1

Supplementary Material 2

## Data Availability

All data generated or analyzed during this study are included in this published article.
