# Supplementary material for: Nonwoven fabric coated with cerium oxide nanoparticles for viral inactivation and transmission Inhibition
Source: Sci Rep. 2025 Mar 25;15:10340. doi: 10.1038/s41598-025-94199-4 (PMC11937568; doi:10.1038/s41598-025-94199-4)
Supplement: Supplementary file 1 — Supplementary Material 1 [file 41598_2025_94199_MOESM1_ESM.pdf]

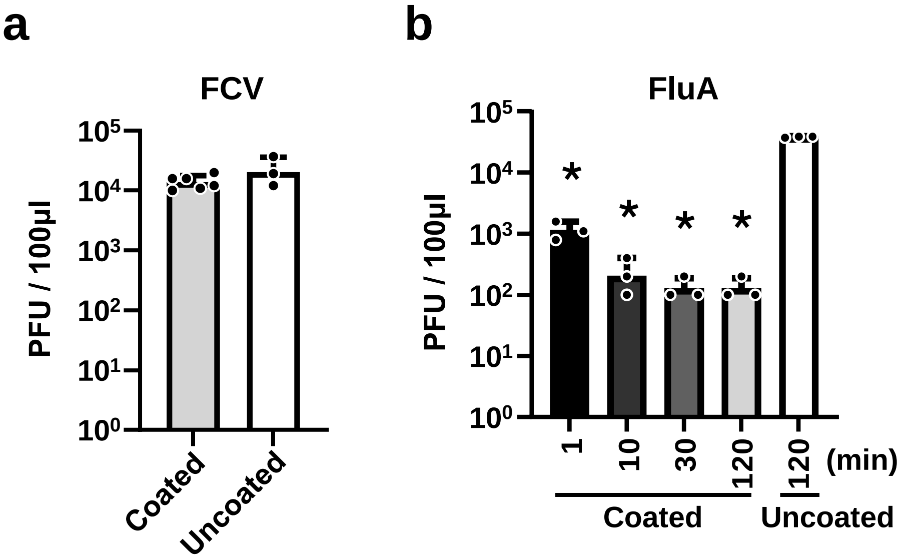

Fig. S1 Evaluation of nonwoven fabrics treated only with the coating agent (without BA-CeO_2)

To verify the antiviral inhibitory activity of the coating agent itself, we tested the fabrics in coated (nonwoven fabrics treated only with the coating agent, excluding BA-CeO_2) and uncoated (untreated nonwoven fabrics) conditions. (a) FCV-containing media were placed on coated or uncoated nonwoven fabrics and collected after 2 h to measure viral titers according to ISO 18184 guidelines. (b) Influenza virus-containing media were placed on coated or uncoated nonwoven fabrics, collected at each time point, and viral titers were measured. The bars represent the mean viral titer observed after each incubation time from three or six independent experiments. Error bars indicate standard deviation. Viral titers were measured using the plaque assay method. Statistical significance was analyzed using the Mann–Whitney U test or a one-way ANOVA followed by Dunnett's multiple comparison test, with $p < 0.05$ being considered significant. *$p < 0.05$ (FCV coated vs. uncoated: $p = 2.74 \times 10^{-1}$; FluA uncoated at 1 min: $p = 5.00 \times 10^{-6}$; FluA uncoated at 10 min: $p = 1.21 \times 10^{-7}$; FluA uncoated at 30 min: $p = 5.38 \times 10^{-8}$; FluA uncoated at 120 min: $p = 5.38 \times 10^{-8}$).
