# Supplementary material for: Nonwoven fabric coated with cerium oxide nanoparticles for viral inactivation and transmission Inhibition
Source: Sci Rep. 2025 Mar 25;15:10340. doi: 10.1038/s41598-025-94199-4 (PMC11937568; doi:10.1038/s41598-025-94199-4)
Supplement: Supplementary file 2 — Supplementary Material 2 [file 41598_2025_94199_MOESM2_ESM.pdf]

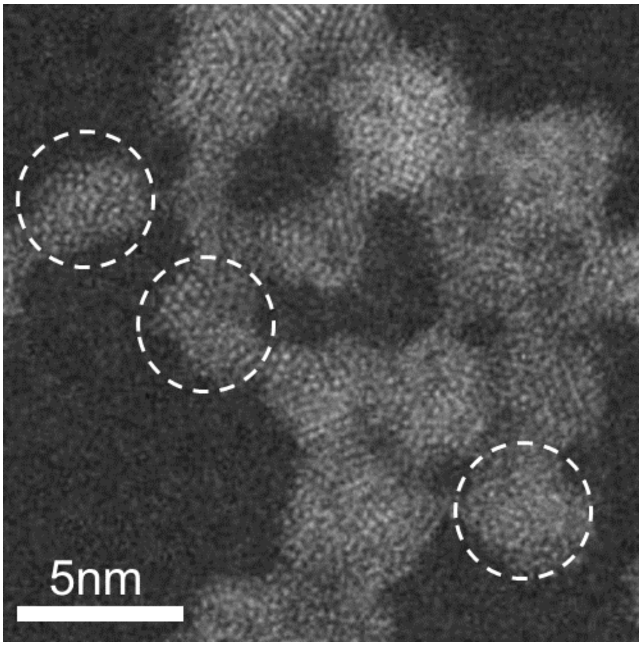

Fig. S2 TEM image of nanoparticles
White dotted circles highlight representative nanoparticles. These secondary particles are composed of primary particles approximately 2-3 nm in size. Similar structures can be observed throughout the field of view.
